# Postnatal Maturation of the Glomerular Filtration Rate in Conventional Growing Piglets As Potential Juvenile Animal Model for Preclinical Pharmaceutical Research

**DOI:** 10.3389/fphar.2017.00431

**Published:** 2017-06-29

**Authors:** Elke Gasthuys, Mathias Devreese, Joske Millecam, Stanislas Sys, Katrien Vanderperren, Joris Delanghe, Johan Vande Walle, Marjolein Heyndrickx, Siska Croubels

**Affiliations:** ^1^Department of Toxicology, Pharmacology and Biochemistry, Faculty of Veterinary Medicine, Ghent UniversityMerelbeke, Belgium; ^2^Department of Internal Medicine and Clinical Biology of Large Animals, Faculty of Veterinary Medicine, Ghent UniversityMerelbeke, Belgium; ^3^Department of Veterinary Medical Imaging and Small Animal Orthopedics, Faculty of Veterinary Medicine, Ghent UniversityMerelbeke, Belgium; ^4^Department of Clinical Chemistry, Microbiology and Immunology, Faculty of Medicine and Health Sciences, Ghent UniversityGhent, Belgium; ^5^Department of Pediatrics and Medical Genetics, Safepedrug, Faculty of Medicine and Health Sciences, Ghent UniversityGhent, Belgium; ^6^“Centre for X-ray Tomography” of the Ghent University/Radiation Physics-Department of Physics and Astronomy, Faculty of Sciences, Ghent UniversityGhent, Belgium

**Keywords:** piglet, glomerular filtration rate, iohexol, creatinine, pediatric animal model

## Abstract

Adequate animal models are required to study the preclinical pharmacokinetics (PK), pharmacodynamics (PD) and safety of drugs in the pediatric subpopulation. Over the years, pigs were presented as a potential animal model, since they display a high degree of anatomical and physiological similarities with humans. To assess the suitability of piglets as a preclinical animal model for children, the ontogeny and maturation processes of several organ systems have to be unraveled and compared between both species. The kidneys play a pivotal role in the PK and PD of various drugs, therefore, the glomerular filtration rate (GFR) measured as clearance of endogenous creatinine (Jaffe and enzymatic assay) and exo-iohexol was determined in conventional piglets aging 8 days (*n* = 16), 4 weeks (*n* = 8) and 7 weeks (*n* = 16). The GFR data were normalized to bodyweight (BW), body surface area (BSA) and kidney weight (KW). Normalization to BSA and KW showed an increase in GFR from 46.57 to 100.92 mL/min/m^2^ and 0.49 to 1.51 mL/min/g KW from 8 days to 7 weeks of age, respectively. Normalization to BW showed a less pronounced increase from 3.55 to 4.31 mL/min/kg. The postnatal development of the GFR was comparable with humans, rendering the piglet a convenient juvenile animal model for studying the PK, PD and safety of drugs in the pediatric subpopulation. Moreover, to facilitate the assessment of the GFR in growing piglets in subsequent studies, a formula was elaborated to estimate the GFR based on plasma creatinine and BW, namely eGFR =1.879 × BW^1.092^$/P_{\mathrm{cr}}^{0.600}$.

## Introduction

One of the key components of the optimization of clinical trials is the development and validation of appropriate preclinical animal models showing a high degree of anatomical and physiological resemblances to humans. In this respect, over the years, the interest has grown to use adult pigs as translational animal models in biomedical research (Bode et al., 2010; Helke and Swindle, 2013; Gasthuys et al., 2016). The considerable anatomical and physiological similarities with humans render the adult pig a valuable alternative to rodents, rabbits, dogs and non-human primates for preclinical pharmaceutical research. In contrast, the suitability of growing piglets as a preclinical model for the pediatric population still needs to be evaluated. In order to contribute to the development of such a porcine model, the maturation processes of several organ systems involved in the disposition of drugs, such as the kidney, as well as a comparison with the human pediatric subpopulation need to be determined. To date, most of the research papers describing the renal function in piglets only focus on one age category using one or more biochemical markers (McCane and Widdowson, 1956; Frennby et al., 1997; Hansen et al., 1997; Bauer et al., 2000). Literature about the ontogeny of the GFR in piglets is rather scarce. Friis (1979) (Danish Landrace breed) and Kaskel and Kleinman (1976) (breed not mentioned) described the postnatal development of the GFR measured as inulin clearance in piglets aging 1–62 and 14–40 days, respectively. An increase in GFR indexed to BW and KW was noted, but more extensive research should be done to further elucidate if the piglet is a suitable preclinical model to study the renal function of the pediatric subpopulation.

The determination of the GFR in the pediatric subpopulation is pivotal for preclinical PK, PD and safety studies, e.g., to determine age-dependent dosing schedules for drugs excreted by and/or acting on the kidney, to evaluate drug toxicity, etc. Moreover, the assessment of the GFR is considered the best overall index of glomerular function, although renal function is more than just glomerular filtration. The GFR can be measured with endogenous and exogenous filtration markers, such as creatinine, cystatin C, urea, inulin, chromium^51^-labeled ethylene diamine tetraacetic acid, ^125^I-iothalamate and iohexol (Hood et al., 1971; Stevens et al., 2006; McPhersen and Pincus, 2011). Clearance of inulin is the gold standard to determine the GFR, but this technique is not routinely applicable as it requires intravenous infusion, timed urine collections and expensive inulin measurements (Herget-Rosenthal et al., 2007). Therefore, in practice, GFR is often estimated from the serum concentrations of endogenous creatinine for both males and females. Notwithstanding that the determination of this marker has multiple confounding factors such as body composition, age, dietary protein intake, chronic illness and interferences with creatinine assays (e.g., ketones, glucose, bilirubin, catecholamines) (Stevens et al., 2006; McPhersen and Pincus, 2011). Various equations were established to circumvent these confounding factors and have been developed to estimate the GFR (Modification of Diet in Renal Disease, Chronic Kidney Disease Epidemiology Collaboration formula) or creatinine clearance (Cockroft and Gault) in adults (Cockcroft and Gault, 1976; Levey et al., 1999; Levey et al., 2009). However, these equations are not suitable for the pediatric subpopulation. Therefore, alternative equations were developed, of which the revised Schwartz formula [0.413 × height (cm)/serum creatinine (mg/dL)] is the most widely used (Schwartz et al., 2009).

The primary aim of the current study was to define the maturation of the GFR in growing conventional piglets aging 8 days, 4 and 7 weeks. For comparative purposes, the GFR was determined as the clearance of both the conventional marker endogenous creatinine (both Jaffe and enzymatic method), and the alternative exogenous marker exo-iohexol, since iohexol is not secreted, metabolized nor reabsorbed by the kidney. Furthermore, the porcine and human postnatal development of GFR was compared. The secondary aim of the present study was to establish a formula to estimate the porcine GFR based on *P*_cr_ and BW, similar to the formula developed by Schwartz et al. (2009) for children. This equation would simplify the determination of the GFR in growing piglets and can be a useful tool in future preclinical porcine studies.

## Materials and Methods

### Animals

The current study was conducted with consent of the ethical committee of the Faculty of Veterinary Medicine and Bioscience Engineering of Ghent University (EC2014/184). Care and use of animals was in full compliance with the most recent national legislation (Belgian Royal Decree, 2013) and European Directive (Anonymous, 2010, Directive 2010/63/EU). Forty-eight healthy stress resistant male piglets aging 8 days (*n* = 16), 4 weeks (*n* = 8), 7 weeks (*n* = 16) and 6 months (*n* = 8) were obtained (Belgian landrace × large white, Seghers Hybrid^®^, Wuustwezel, Belgium). The piglets of 8 days, 4 and 7 weeks of age were included in both the GFR study and for the estimation of the creatinine formula, the 6-month-old piglets only for the elaboration of the creatinine formula. The 8-day-old piglets were housed individually in Rescue Decks (0.9 m × 1.40 m) (Provimi, Rotterdam, The Netherlands), whereas the older age categories were housed individually in standard pig stables (2.30 m × 2.40 m). Throughout the experiment, the animals had free access to milk (8 days; Rescuemilk^®^, Provimi), feed (4 and 7 weeks; Piggistart Opti^®^, 6 months: Optivo Pro^®^, Aveve, Leuven, Belgium) and drinking water. Stable temperature was 23.2 ± 0.56°C and a natural light scheme was provided by transparent windows. During the experiments, the stables were enriched with hanging chains, rubber toys and balls of different sizes. The environmental enrichment was rotated daily. One day after arrival, a urine collection bag (Esteem Synergy^®^ Uro, 48 mm, ConvaTec, Belgium) was adhered around the prepuce of the piglets (except the 6-month-old pigs) and a double-lumen catheter was inserted in the jugular vein (all age groups) of the piglets according to the surgical procedures in Gasthuys et al. (2017).

### Experimental Design

One day after the surgery, endogenous creatinine concentrations were determined by collecting 1 mL of blood at three different time points, namely 10 am, 2 pm and 6 pm (K_3_EDTA tubes, Vacutest^®^, Kima, Italy) and collecting urine at three different time intervals (1 h), namely 10–11 am, 2–3 pm and 6–7 pm. These three different time points/intervals were chosen to compare the influence of time of the day on the creatinine concentrations. The blood samples were immediately transferred on ice and centrifuged within 2 h (2095 × *g* for 10 min at 4°C). Plasma was aliquoted and stored at -20°C until analysis (all age groups within 1 week except the 4 weeks within 1 month). The urine bags were weighted to determine the volume and an aliquot was stored at -20°C until analysis (all age groups within 1 week except the 4 weeks within 1 month). The following day, a single dose of 64.7 mg/kg BW of iohexol (0.1 mL/kg BW, Omnipaque^®^ 300 mg I/mL, GE Healthcare, Belgium) was administered intravenously using the proximal lumen of the jugular catheter. Blood samples (1 mL) were collected from the distal lumen of the catheter at 0 min (just before administration), and at 5, 15, 30, 60, 120, 180, 360, 480, 600, and 1440 min post administration. The samples were drawn into 9 mL K_3_EDTA collection tubes (Vacutest^®^), immediately kept on ice and centrifuged for 10 min at 2095 × *g* within 2 h after blood collection. Supernatant was aliquoted, frozen and stored at -20°C until analysis (all age groups within 1 month). After the experiment, euthanasia was performed by administering an overdose of sodium pentobarbital (sodium pentobarbital 20%^®^, Kela, Belgium) through the proximal lumen of the jugular catheter. Both kidneys were collected for further analysis (see Laboratory Analyses for Creatinine and Exo-iohexol Quantification).

### Laboratory Analyses for Creatinine and Exo-iohexol Quantification

Creatinine concentrations were determined in plasma and urine using both a kinetic rate-blanked Jaffe compensated assay for reactive proteins according to the manufacturer’s instructions (Wuyts et al., 2003) and an enzymatic creatinine assay using the Creatinine Plus method (Goren et al., 1986) (Laboratory of Clinical Chemistry, Ghent University Hospital, Ghent, Belgium). The GFR was calculated using the following equation: clearance of creatinine (mL/min)= (U_cr_^∗^U_vol_)/(P_cr_^∗^T_min_), where *U*_cr_ is urinary creatinine concentration in mg/dL, *U*_vol_ in volume of urine in mL, *P*_cr_ is plasma creatinine concentration in mg/dL, and *T*_min_ is timed urine collection interval in minutes. Serum total protein was assayed using the biuret method with commercial available reagents (Roche) (Laboratory of Clinical Chemistry) to correct the Jaffe results for the protein error in the alkaline picrate reaction (Speeckaert et al., 2012). When the total plasma protein content was lower than 75 g/L a correction factor was implemented to calculate the GFR (corrected *P*_cr_ Jaffe = (*P*_cr_ + 0.23)-[(0.23/75)^∗^total protein content)]. To determine possible outliers in the urinary Jaffe results, the 24h-creatinine content in urine (creatinine load) was calculated [U_cr_^∗^(U_vol_/100)/24^∗^(T_min_/60)]. The 24h-creatinine content was divided by the BW and potential outliers were determined after visual inspection of the data.

Exo-iohexol plasma concentrations were determined by an in-house validated HPLC-UV (De Baere et al., 2012) (Laboratory of Pharmacology and Toxicology, Ghent University, Merelbeke, Belgium). In brief, 25 μL of internal standard (1 mg/mL in methanol, iohexol impurity J, Chemical Reference Substance of the European Pharmacopoeia, Strasbourg, France) was added to each sample (100 μL of plasma and 100 μL of HPLC water), followed by a deproteinization step with trifluoroacetic acid and centrifugation of the plasma sample. The supernatant was transferred to an autosampler vial and 50 μL was injected onto the HPLC-UV instrument (Thermo Separations Products, Thermo Scientific, The Netherlands). The AUC_0-inf_ was determined by the logarithmic trapezoidal method with extrapolation to infinity. The plasma exo-iohexol clearance was calculated by dividing the dose of exo-iohexol (determined by the ratio of exo-iohexol and endo-iohexol in plasma) by this AUC_0-inf_.

Both the creatinine as the exo-iohexol clearances were normalized and indexed to BW (mL/min/kg), BSA (mL/min/m^2^) and KW (mL/min/g). The body surface of the piglets (8 days, 4 and 7 weeks) was imaged using a four-slice helical CT-scanner, where the piglets were placed in ventral recumbency (Lightspeed QC/I, GE Medical systems, New York City, NY, United States). The CT-scans were processed using VGStudioMax (Volume Graphics GmbH, Heidelberg, Germany) and the BSA was determined after visualization and selection of the body surface in 3D (UGCT-Department of Physics and Astronomy, Ghent University, Ghent, Belgium). To determine the KW, both piglets’ kidneys were removed immediately after euthanasia, cleaned from excess blood using compresses and weighted.

### eGFR Creatinine Formula

For the elaboration of the porcine GFR estimation formula (eGFR), regression models were calculated to predict the GFR based on *P*_cr_, BW, age and time of blood sampling (SPSS 23, IBM, United States). Four different age categories were included, e.g., 8 days, 4 weeks, 7 weeks and 6 months. The general regression model was drafted after log-transformation of the continuous variables (*P*_cr_, BW and age):

$$\begin{array}{c} \;\begin{array}{c} \mathrm{Log}(\mathrm{eGFR})\;=a+b\log(P_{\mathrm{cr}})+c\log(\mathrm{BW})+d\log(\mathrm{age})\;\\ \;+e\log{\left(\mathrm{age}\right)}^{*}\log(P_{\mathrm{cr}})+f\log{\left(\mathrm{age}\right)}^{*}\log(\mathrm{BW}) \end{array}\; \end{array}$$

Regression analyses (SPSS 23, IBM, United States) were performed in stepwise order (inclusion criterion: *p* < 0.05; exclusion criterion: *p* > 0.10). Regression analyses were performed based either on the *P*_cr_ values at a specific time of blood sampling (*P*_cr_10am, *P*_cr_2pm, and *P*_cr_6pm), or on the mean *P*_cr_ over the different times of blood sampling. The appropriateness of the GFR estimating equation was assessed for each equation by determining the adjusted *R*^2^, which reflects the percentage of variability in GFR explained by the regression equation. Moreover, the relative accuracy was determined as the percentage of eGFR values falling within 10 and 30% of the measured GFR.

### Statistical Analysis

Statistical analysis of creatinine and exo-iohexol clearance was carried out separately for the different normalization methods and was performed on the log-transformed GFR data (SPSS 23, IBM, United States). The global effect and interaction of the factors age and clearance technique (creatinine determined by Jaffe and enzymatic method and exo-iohexol) was analyzed in a mixed model with repeated measurements on technique. For each of the normalization methods, the interaction between age and technique revealed statistically significant differences. Within each technique, the effect of age was further analyzed using one-way analysis of variance (ANOVA). Within each age, the effect of technique was further analyzed in a mixed model with repeated measurements on technique. *Post hoc* comparisons test was performed according to Bonferroni. The level of significance was set at 0.05.

## Results

All piglets survived catheterization of the jugular vein without any complications. Two out of 16 piglets aging 8 days were withdrawn from the study (after surgery) due to low BW (<1.50 kg). **Table 1** summarizes the descriptive statistics of the piglets included in the study with respect to BW, BSA and KW at the age of 8 days, 4 weeks, 7 weeks and 6 months. GFR (mean ± SD) values indexed to BW, BSA and KW for the different age categories are presented in **Table 2**. No influence of time of the day on the *P*_cr_ was observed, consequently the mean *P*_cr_ was used to calculate the GFR for Jaffe and the enzymatic method. The mean renal clearance of exo-iohexol indexed to BSA showed a significant increase (*p* < 0.05) from 46.57 mL/min/m^2^ at 8 days of age to 100.92 mL/min/m^2^ at 7 weeks of age. The increase in GFR was also significant for the youngest age categories (8 days versus 4 weeks, 8 days versus 7 weeks) for the conventional creatinine measurement methods (Jaffe and enzymatic), but did not differ significantly between 4 and 7 weeks of age. When GFR was indexed to BW, only the exo-iohexol clearance of the youngest age category was significantly lower compared to the older age categories. Normalization of GFR to KW showed the same trend for the different clearance techniques, namely significantly lower GFR values for the 8-day-old piglets in comparison with the 4- and 7-week-old piglets. Determinations of the GFR with the three different techniques were statistically equivalent, except at the age of 4 weeks, where the clearance of creatinine (Jaffe and enzymatic) was significantly higher than the clearance of exo-iohexol.

**Table 1:** Descriptive statistics of the piglet study population. Values are mean ± SD.

Parameter	8 days	28 days (4 weeks)	49 days (7 weeks)	180 days (6 months)
Number of piglets	14	8	16	8
Bodyweight (kg)	2.01 ± 0.43	10.0 ± 1.69	13.9 ± 2.74	112.88 ± 9.11
Body surface area (m^2^)	0.16 ± 0.02	0.46 ± 0.05	0.59 ± 0.08	2.42 ± 0.13
Kidney weight (g)	14.0 ± 2.94	20.25 ± 3.30	38.0 ± 3.16	121.24 ± 20.97
Plasma creatinine (mg/dL)	0.43 ± 0.08	0.46 ± 0.11	0.45 ± 0.10	0.99 ± 0.11
Total plasma protein (g/L)	28.4 ± 5.60	31.7 ± 5.59	31.6 ± 6.39	49.2 ± 6.83

**Table 2:** Glomerular filtration rate measurements (GFR, mean ± SD) with Jaffe, enzymatic creatinine assay and exo-iohexol in growing piglets aging 8 days (*n* = 14), 4 weeks (28 days, *n* = 8), 7 weeks (49 days, *n* = 16).

Age	GFR enzymatic			GFR Jaffe			GFR exo-iohexol		
Days	Indexed	Indexed	Indexed	Indexed	Indexed	Indexed	Indexed	Indexed	Indexed
	to BW^∗1^	to BSA^∗2^	to KW^∗3^	to BW^∗1^	to BSA^∗2^	to KW^∗3^	to BW^∗1^	to BSA^∗2^	to KW^∗3^
8	3.94 ± 1.45	51.55 ± 20.46^c,d^	0.54 ± 0.25^h,i^	3.53 ± 0.88	46.24 ± 12.87^e,f^	0.49 ± 0.17^j,k^	3.55 ± 1.01^a,b^	46.57 ± 13.88^g^	0.49 ± 0.16^l,m^
28	5.06 ± 0.91^1^	111.00 ± 17.71^c,3^	2.40 ± 0.35^h,5^	4.76 ± 1.24^2^	104.88 ± 26.96^e,4^	2.29 ± 0.63^j,6^	3.10 ± 0.53^a,1,2^	67.36 ± 11.54^g,3,4^	1.43 ± 0.30^l,5,6^
49	4.74 ± 2.15	108.70 ± 47.20^d^	1.56 ± 0.66^i^	4.83 ± 2.48	112.40 ± 55.56^f^	1.63 ± 0.80^k^	4.31 ± 0.70^b^	100.92 ± 20.84^g^	1.51 ± 0.47^m^

*The GFR is indexed to bodyweight, body surface area and kidney weight. Results with the same alphabetical character superscript are considered significant different (*p* < 0.05) between the different age categories within one technique. Results with the same numerical character superscript are considered significant different (*p* < 0.05) between the different techniques used within one age category. ^∗1^, mL/min/kg; ^∗2^, mL/min/m^*2*^; ^∗3^, mL/min/g.*

A regression model was determined to predict the GFR based on *P*_cr_, BW, age and time of blood sampling (**Table 3**). The inclusion of the continuous variables *P*_cr_ and BW was significant (*p* < 0.05) and the variability in GFR that can be predicted by the equation was 97.7%. No improvement of GFR estimation was observed by including the time of blood sampling (for example *P*_cr_mean versus *P*_cr_2pm, **Table 3**) and age (model I) within the regression model. Consequently, these variables were omitted from the equation. After adjustment of the full regression model, an adapted significant regression equation (*P*_cr_mean, model I) was found:

**Table 3:** Properties (adjusted R^2^ and the relative accuracy) of eGFR equations derived from coefficients of different regression models.

	eGFR = a × BW^b^/*P*_cr_^c^					
	a	b	c	R^2^ (%)	% of eGFR within 30% of GFR	% of eGFR within 10% of GFR
**P_cr_mean**						
Age mean	Age mean	–	–	0.0	72.7	34.1
Model I	1.879	1.092	–0.600	97.7	90.9	36.4
**P_cr_2pm**						
Age mean	Age mean	–	–	0.0	72.7	34.1
Model I	2.339	1.052	–0.442	97.6	90.7	34.9

*Age was not included within the regression model, because the coefficients d,e,f were not significant (*p* < 0.05) for both models [*P*_*cr*_mean: *p* = 0.408 (d), *p* = 0.114 (e), *p* = 0.565 (f); *P*_*cr*_2pm: *p* = 0.087 (d), *p* = 0.338 (e), *p* = 0.095 (f)]. P_cr_ = plasma creatinine concentration; eGFR = estimated glomerular filtration rate; BW = bodyweight; R^*2*^ = coefficient of determination.*

$$\begin{array}{c} \mathrm{eGFR}\;=\;\frac{1.879\times BW^{1.092}}{P_{\mathrm{cr}}^{\;0.600}} \end{array}$$

## Discussion

Gaining insight in the maturational changes of the kidney function is important for properly conducting preclinical animal trials for pediatric drug development. The current study describes the postnatal maturation of the GFR determined with endogenous creatinine (Jaffe and enzymatic method) and exo-iohexol in growing piglets. It was opted to choose conventional piglets instead of minipigs, since the use of neonatal minipigs for repetitive urine and blood collections remains challenging. It was not possible to determine the GFR with endogenous creatinine in female and 6-month-old piglets, since an optimal non-invasive urine collection technique is still lacking (Gasthuys et al., 2017). Therefore, this study focused on male piglets aging 8 days to 7 weeks. Scaling of the GFR to a standard reference is necessary to compare GFR values among populations. In human clinical practice, the GFR is traditionally normalized to a standardized BSA (1.73 m^2^), since BSA correlates closely with kidney size (McIntosh et al., 1928). However, the appropriateness of normalizing to BSA is arguably in the pediatric subpopulation, as the formula to calculate the BSA tends to underestimate the true BSA and standardization to GFR/1.73 m^2^ was 20% lower than GFR/12.9 L extracellular fluid volume (Geddes et al., 2008). In veterinary clinical practice, the GFR is normalized to BW, nonetheless this normalization was outdated in human clinical practice, because BW is mainly influenced by body fat content which has a limited influence on PK and PD for most drugs (Bird et al., 2003). Scaling to KW should be more appropriate, but is not clinically applicable (Schwartz and Work, 2009). In the current study, all GFR values were adjusted for BW, BSA or KW. When indexed to BSA and KW, a statistical significant increase in GFR was observed. The increase in GFR indexed to BW was less pronounced than the GFR indexed to KW and BSA. Kaskel and Kleinman (1976) and Friis (1979) also measured the GFR as inulin clearance in growing piglets indexed to KW (Kaskel and Kleinman, 1976; Friis, 1979) and BW (Friis, 1979). The increasing GFR in those studies was similar to the trends observed in the current study. In humans, the GFR is 2–4 mL/min/1.73 m^2^ at birth, increases rapidly to 39–47 mL/min/1.73 m^2^ during the first 2 weeks of life, continues rising to 58–103 mL/min/m^2^ during the first year of life and reaches adult values (127 mL/min/1.73 m^2^) at the age of 2 years (Guignard et al., 1975; Heilbron et al., 1991). The age correlations between humans and pigs proposed by Gad (2007) were considered here to compare the GFR between humans and piglets, namely neonate (human: 0–28 days, pig: 0–15 days), infant (human: 1–23 months, pig: 2–4 weeks), child (human: 2–12 years, pig: 4–14 weeks) and adolescent (human: 12–16 years, pig: 4–6 months). The postnatal maturation of GFR indexed to BSA in the current study (e.g., measured with exo-iohexol: 8 days: 46.57 mL/min/m^2^, 4 weeks: 67.36 mL/min/m^2^, and 7 weeks: 100.92 mL/min/m^2^) was in accordance with the increasing trend observed in humans.

It is generally accepted in pediatric clinical practice, that the use of the endogenous clearance marker creatinine to measure GFR is less accurate in comparison with the exogenous clearance marker iohexol (Filler et al., 2014). However, the data demonstrated in the present study show a good agreement between the three techniques, except at the age of 4 weeks. The differences between the techniques might be explained by the fact that creatinine in urine is the result of both glomerular filtration and tubular secretion, leading to an overestimation of the creatinine clearance (Carlier et al., 2015). The presence of tubular secretion function, is even more documented by administrating p-aminohippuric acid (PAH) to piglets, since the tubular functional capacity can be assessed by plasma clearance of PAH (Tett et al., 2003). Another possible explanation is the difference in storage period between the 4 weeks (1 month) and the other age groups (1 week), leading to confounded plasma and/or urine creatinine concentrations. There are also some disadvantages to the measurement of GFR by endogenous creatinine clearance in piglets, rendering this technique less reliable. First, ideally urine collection should be done over a 24h-period, but the collection of 24h-urine is difficult to perform, whereby a sparse sampling procedure was needed during the experiments. To tackle this problem in the current study, the creatinine load was calculated and outliers were excluded from the dataset. Second, the determination of plasma creatinine with Jaffe is not reliable when the total plasma protein content is lower than 75 g/L. Therefore, the total plasma protein concentration must be measured and a correction factor must be implemented to calculate the GFR, which was done in the present study. Third, Hansen et al. (1997) suggested the possibility that transport of endogenous creatinine by the renal tubules varies between breeds, rendering the endogenous creatinine determination less accurate. Iohexol is a more reliable marker, since it only needs blood sampling and no urine collection, and the determination of iohexol is less sensitive to confounding factors.

To date, the determination of the true GFR is time-consuming, costly and labor-intensive. Because GFR is difficult to measure in clinical practice, different equations for humans were developed to predict GFR using endogenous creatinine (e.g., Schwartz et al., 2009). Unfortunately up till now, no equations are available for piglets. Therefore, a formula was determined to estimate the creatinine clearance (eGFR) based on readily available parameters, such as creatinine plasma concentration, BW, age and time of blood sampling. The GFR estimation was based on the enzymatic endogenous creatinine determination, since the values obtained by the Jaffe technique are more affected by interferences, such as the presence of ketones, fructose, glucose, etc., in plasma (Stevens et al., 2006). The enzymatic assay reduced the problem of these interferences, but it should be emphasized that possible variation in calibration of the assays among laboratories may lead to different results. However, this variation is expected to be minimal (Stevens et al., 2006). The presented equation can be a useful tool for preclinical pediatric porcine studies, as well as a tool to determine correct dosage strategies for drugs that are renally excreted in growing piglets.

## Conclusion

The current study determined the maturation of the kidney function in growing piglets, which was comparable to humans, in a search for a more suitable pediatric model than rodents. The values obtained with the different GFR techniques were comparable, except at the age of 4 weeks, although GFR measured by exo-iohexol clearance is preferred. To further proof the usefulness of the piglet as potential animal model, in future research, PK studies of mainly renally excreted drugs should be performed and results should be compared to published data in children. Moreover, an easy-to-apply creatinine equation was developed to estimate the GFR in growing piglets and to provide a useful tool in preclinical conventional porcine studies.

## Author Contributions

EG: performed and coordinated the animal studies, performed the analyses, drafted the manuscript, and approved the final manuscript as submitted. MD: reviewed and revised the manuscript and approved the final manuscript as submitted. JM: performed the animal studies, reviewed and revised the manuscript and approved the final manuscript as submitted. SS: performed the statistical analyses, drafted the manuscript, reviewed and revised the manuscript and approved the final manuscript as submitted. KV: performed the CT-scans, reviewed and revised the manuscript and approved the final manuscript as submitted. JD: performed the laboratory analyses, reviewed and revised the manuscript and approved the final manuscript as submitted. JVW: reviewed and revised the manuscript and approved the final manuscript as submitted. MH: performed the CT-scan analysis, reviewed and revised the manuscript and approved the final manuscript as submitted. SC: reviewed and revised the manuscript and approved the final manuscript as submitted.

## Conflict of Interest Statement

The authors declare that the research was conducted in the absence of any commercial or financial relationships that could be construed as a potential conflict of interest.

## Abbreviations

AUC: Area under the plasma concentration-time curve
BSA: Body Surface Area
BW: Bodyweight
eGFR: Estimated Glomerular Filtration Rate
GFR: Glomerular Filtration Rate
HPLC-UV: High-Performance Liquid Chromatography Method with Ultraviolet Detection
KW: Kidney Weight
*P*_cr_: Plasma creatinine concentration
PD: Pharmacodynamic
PK: Pharmacokinetic
*T*_min_: Timed urine collection intervals
*U*_cr_: Urinary creatinine concentration
*U*_vol_: Volume of urine.
